# Improving polygenic risk prediction of renal function by removing biomarker-specific effects

**DOI:** 10.21203/rs.3.rs-6329094/v1

**Published:** 2025-04-03

**Authors:** Megan Shuey, Jiawen Du, Quan Sun, Laura Zhou, Nora Franceschini, Nancy J. Cox, Yun Li

## Abstract

Biomarkers are frequently used in clinical practice; however, it is essential to consider the genetics that may independently impact their baseline levels in-turn impacting interpretation of results. For example, estimated glomerular filtration rate (eGFR) equations are based on two biomarkers, cystatin C and creatinine, and widely employed in clinical practice. In this work we demonstrate how genetics of the underlying biomarkers impact measurement variability and may explain some of the discrepancies among eGFR equations. To do this we used shared genetic-architecture to identify “shared” (renal-specific) and “biomarker-specific” regions of the genome. The shared polygenic risk score (PRS) explained 60% more of the variability in the most-common creatinine-derived eGFR estimates than either the biomarker-specific PRS or a PRS including all regions. Our findings highlight the necessity of considering biomarker-specific genetics when constructing PRS for eGFR and other biomarker-derived clinical risk estimates.

## Full Text

Biomarkers have emerged as important tools in estimating disease risk or monitoring progression[1]. Creatinine and cystatin C are two of the most widely studied biomarkers and used in clinical practice to estimate renal function as part of estimated glomerular filtration rate (eGFR) equations.

Despite the utility of these biomarkers the derived eGFR equations demonstrate inherent limitations some of which reflect the biomarkers themselves[2]. For example, both biomarkers exhibit substantial heritability which may mask some of the dynamic changes expected with decline potentially impacting early detection of renal failure. This high heritability may also reflect non-renal influences of endogenous biomarker levels that are often unaccounted for in estimating equation. This is particularly true for cystatin C levels that are impacted by diabetes, proteinuria, thyroid disease, glucocorticoids, smoking, and inflammation[3-5]. Comparatively, creatinine levels are more often related to age, sex, and muscle mass, adjustments largely reflected in the eGFR equations[3,6]. Despite these limitations, eGFR remains the standard for renal function assessment in clinical practice due to the invasiveness, cost, and complexity of directly measuring GFR[7].

The Chronic Kidney Disease Epidemiology Collaboration eGFR equations for creatine excluding race correction ($eGFRcr^{2021}$), cystatin C (eGFRcys), and combined ($eGFRcr\text{-}cys^{2021}$) are the current standard[8,9]. Earlier equations ($eGFRcr^{2009}$ and $eGFRcr\text{-}cys^{2012}$) including race adjustments are still used in older datasets[10], complicating genetic studies reliant on summary-level data. Using UK BioBank (UKB), we assessed the variability of eGFR estimates across equations[11] in four population groups, African (AFR, N=9,216), European (EUR, N=449,300), East Asian (EAS, N=2,532) and South Asian (SAS, N=9,629) (**Methods**)[12]. Comparisons within each ancestral group demonstrated inconsistent estimates (**Fig.1**). For example, compared to creatinine estimates ($eGFRcr^{2021}$ and $eGFRcr\text{-}cys^{2021}$), eGFRcys provided lower mean values and a less skewed distribution in EAS, EUR, and SAS. These patterns were completely opposite in AFR (**Fig.1a-c**). Similar discrepancies are observed comparing the old and new equations based on the same biomarker with or without race correction (**Fig.1d,e**). These results demonstrate that eGFR estimates vary within different groups and across equations.

We hypothesized that these inconsistencies would be reflected as discrepancies in genetic associations. Therefore, we performed separate genome-wide association studies (GWASs) for the five eGFR equations in 441,906 UKB EUR individuals (**Methods**). While, the GWASs had similar genetic inflation factors and heritability estimates (**Extended Data Table 1**) consistent with previous publications[13], the genetic correlation analyses showed significant difference. The lowest genetic correlation was between eGFRcys and eGFRcr (0.587,eGFRcr$^{2021}$; 0.590,eGFRcr$^{2009}$). The combined equation, eGFRcr-cys showed high genetic correlation with both, eGFRcys(~0.9) and eGFRcr(~0.8) (**Extended Data Table 2)**.

To understand the potential drivers of variable correlation estimates, we evaluated specific loci. The region overlapping the human cystatin C gene (*CST3*) was highly significant in the GWASs for eGFRcys, eGFRcr-cys$^{2021}$ and eGFRcr-cys$^{2012}$ and included the most significant variant rs734801(20:23612791:A:G, hg19, p=1e-6181). This cystatin C specific locus was absent in eGFRcr$^{2021}$ and eGFRcr$^{2009}$ GWAS and the variant was not significant (p=0.122 and 0.158, respectively) supporting that the locus is related to the biomarker instead of eGFR. We observed additional regions with inconsistent impacts between eGFRcr and eGFRcys (**Extended Data Fig 1)**, indicating they were not driven by few specific loci.

Because the discrepancies may reflect differences in the underlying genetic components of the biomarkers, we focused on finding the shared genetic architecture of eGFRs derived from creatinine and cystatin C. Genetic determinants in these shared-regions may reflect eGFR while significant genomic regions unique to a single biomarker likely reflect biomarker-specific genetic effects. We conducted local genetic correlation analyses using LOGODetect[14] with the summary statistics obtained from training GWASs in a subset of UKB EUR individuals (n = 350,000) (**Methods**). We identified 278 genome regions with varying sizes adaptively determined by LOGODetect (**Fig.2a, Methods, Extended Data Fig 2-4**), covering on average 42% of the total genome-wide significant($p<5e-8$) variants of the 5 eGFRs. Variants in the shared regions showed greater consistency in terms of the association with different eGFR equations. When restricted to shared-region variants, the correlation of pairwise -log10p from different eGFR GWASs increased up to 57% compared to all variants. The largest increment of correlation was 0.519 to 0.816 between eGFRcys and eGFRcr$^{2021}$(**Extended Data Fig 5**).

To investigate if variants inside the shared regions better reflect genetic disposition to eGFR and improve PRS prediction, we constructed 3 eGFR PRSs using PRC-CS[15] with 3 variant sets: (1)a benchmark PRS using all variants in eGFR GWAS (n=1,117,353, **Fig.2b, left branch**), (2)a “shared” PRS using only variants in shared-regions (n=17,991, **Fig.2b, middle branch**), and (3)a “biomarker-specific” PRS using variants outside shared-regions and not in high LD($R^2$>0.8) with shared-region variants (**Fig.2b, right branch**) (**Methods**). We then evaluated these three PRSs using $R^2$ increment compared to a baseline, covariatesonly model (**Methods**). Strikingly, our “shared” PRS explained 60% more variation of eGFRcr, compared to either the benchmark or “biomarker-specific” PRS (partial $R^2$=4.44% vs 2.77% or 1.27%)(**Fig.2c, Extended Data Table 3 and 4**). Considering eGFRcr is the most popular estimate for estimating GFR, our “shared” PRS successfully used much fewer variants (17K vs. 1.1M) to attain higher prediction power. The “shared” PRS only reached 38% of performance for eGFRcys compared to benchmark. Due to the nature of cystatin C biology where extrarenal influences are known to impact basal levels, we hypothesized that the low performance is related to other factors that influence the genetic predisposition to these conditions. To assess this, we included adjustments for body mass index (BMI) and a BMI PRS, surrogates for obesity. After adjusting for BMI and BMI PRS, the covariates-only model $R^2$ increased from 0.27 to 0.34 for eGFRcys but stayed the same for eGFRcr (**Extended Data Table 3 and 4**) demonstrating that the addition of BMI and BMI PRS significantly improved prediction of eGFRcys but not eGFRcr or eGFRcr-cys. Because creatine levels are not impacted by adiposity, these findings support that genetic predisposition to non-renal influences impact basal cystatic C levels and may not accurately reflect renal function alone.

To validate these findings, we evaluated the performance of the three PRSs against $eGFRcr^{2021}$ in 70,824 patients of genetically determined European ancestry in Vanderbilt University Medical Center’s BioVU (**Methods**). Consistent with the observations in UKB, the “shared” PRS had a 5.2% improvement in $R^2$ compared to benchmark. While all scores were significantly associated with the $eGFRcr^{2021}$, there was a five-fold increase in significance for "shared" PRS compared to benchmark including all variants (p=8.5e-172 vs. p=3.5e-215). While the biomarker-specific PRS was least significant, p=6.4e-54.

We demonstrated that removing loci related to the biomarker-specific genetics of creatinine and cystatin C can dramatically improve the ability of PRSs to predict eGFR derived from creatine. In doing so, we developed a partitioned “shared” PRS for eGFR that uses variants likely reflecting renal-specific influences instead of the biomarkers. This partitioned PRS has the potential to more holistically represent renal function and reduce biases created by the extrarenal influences related to the biomarkers. Despite the promise of this work, there are limitations. As a proof-of-principal analysis, we restricted to the largest sample population of these biomarkers. We were also not able to assess the ability of the “shared” PRS to improve prediction of directly measured renal function. Future work to consider individuals of heterogeneous ancestries and assessment against measured renal function is important to fully realize the potential to improve eGFR by accounting for “biomarker-specific” genetics.

This work focused on biomarkers used to estimate renal function, however, the principle of considering the potential impacts of biomarker-specific genetics is broadly applicable. While biomarkers can dramatically improve our clinical understanding of disease progression and risk, there are potential limitations in their use and interpretation when their basal levels are dramatically influenced by genetic predisposition. Without adequately considering genetic background, utilization of these clinical biomarkers can result in dramatic health disparities and risk. Another example of the potential consequences of inadequately accounting for genetic predisposition in a biomarker, is white blood cell

(WBC) count and the Duffy-null allele, rs2814778-CC. Individuals of African Ancestry with rs2814778-CC were more likely to receive bone marrow biopsies due to elevated WBCs, yet 97% of the time no hematologic abnormality was identified[16]. A cross-biobank phenome-wide association study of rs2814778-CC similarly identified only significant associations with WBC traits[18]. Taken together, this work and ours suggest that efforts to regress out biomarker-specific genetics may help explain inter-individual variability that is not reflective of specific disease states or outcomes.

## Declarations

**Funding sources:** Research reported in this publication was supported by the National Institutes of Health for the project "Polygenic Risk Methods in Diverse Populations (PRIMED) Consortium," with grant funding for EndoPhenotype InCorporated PRS (National Human Genome Research Institute grant U01HG011720) study site. The content is solely the responsibility of the authors and does not necessarily represent the official views of the National Institutes of Health. The data derived from Vanderbilt University Medical Center are supported by numerous sources, including the NIH-funded Shared Instrumentation Grants S10OD017985 and S10RR025141; National Center for Advancing Translational Sciences CTSA grants UL1TR002243, UL1TR000445, and UL1RR024975; and investigator-led project grants. A full list of VUMC associated funding sources is available at https://victr.vumc.org/biovu-funding/. M.M.S. was supported by K12AR084232. N.F. and Y.L. were supported by R01HL163972.

**Conflicts of interest:** The authors have no conflicts of interest to declare.

## Figures

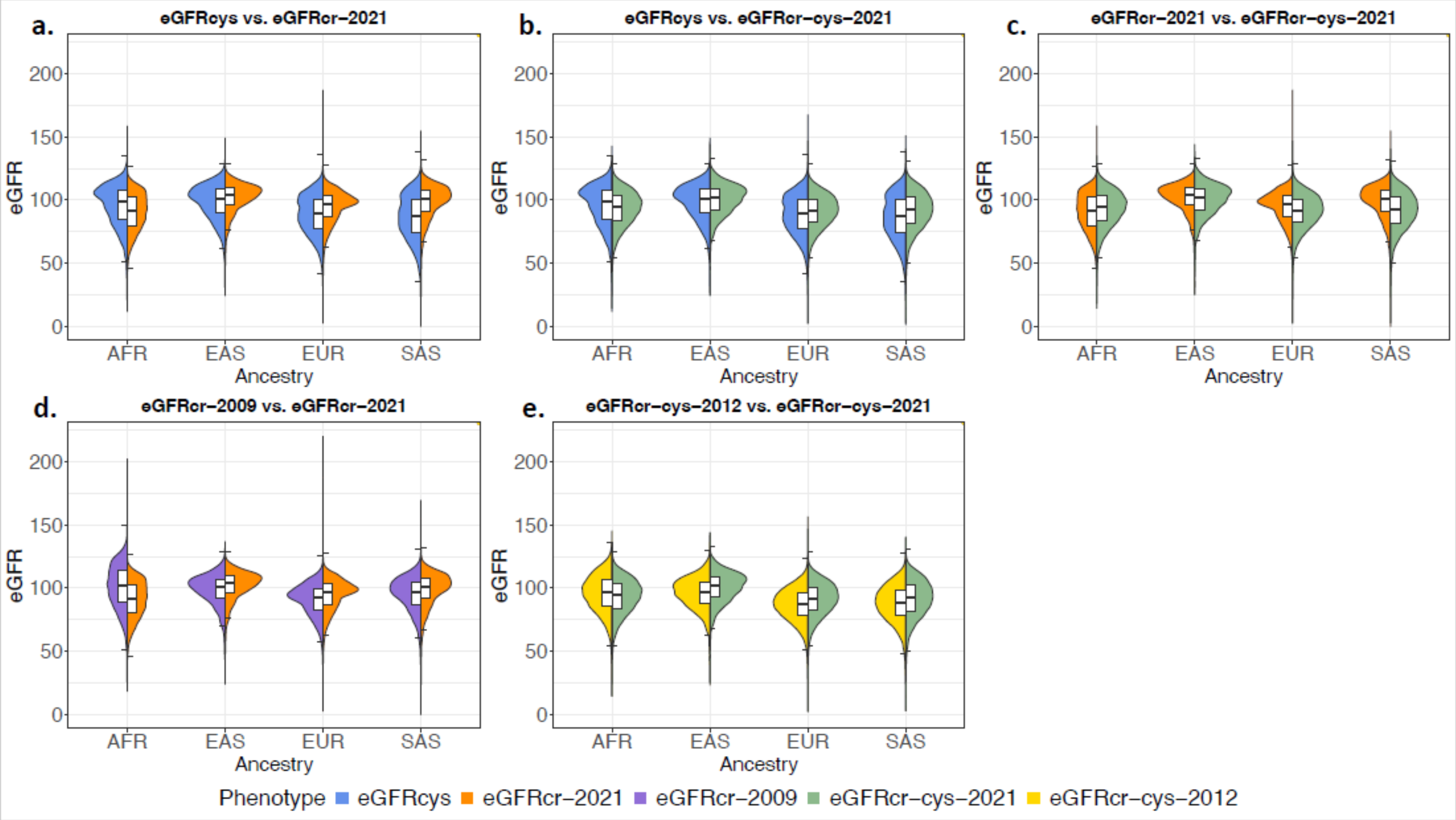

### Figure 1

**Half-half violin plots of distribution of five eGFR values across four ancestry populations. a-c.**Pairwise comparison of eGFRcys, $eGFRcr^{2021}$, and eGFRcr-$cys^{2021}$. In EAS, EUR and SAS, creatinine-based equations provided higher estimates than the combined and cystatin-C based equations. This pattern is reversed in AFR. **d,e.**Comparison of new and old equations($eGFRcr^{2021}$ vs. $eGFRcr^{2009}$; eGFRcr-$cys^{2021}$ vs. eGFRcr-$cys^{2012}$). The new equations provide higher estimates in EAS, EUR and SAS while the old is higher in AFR.

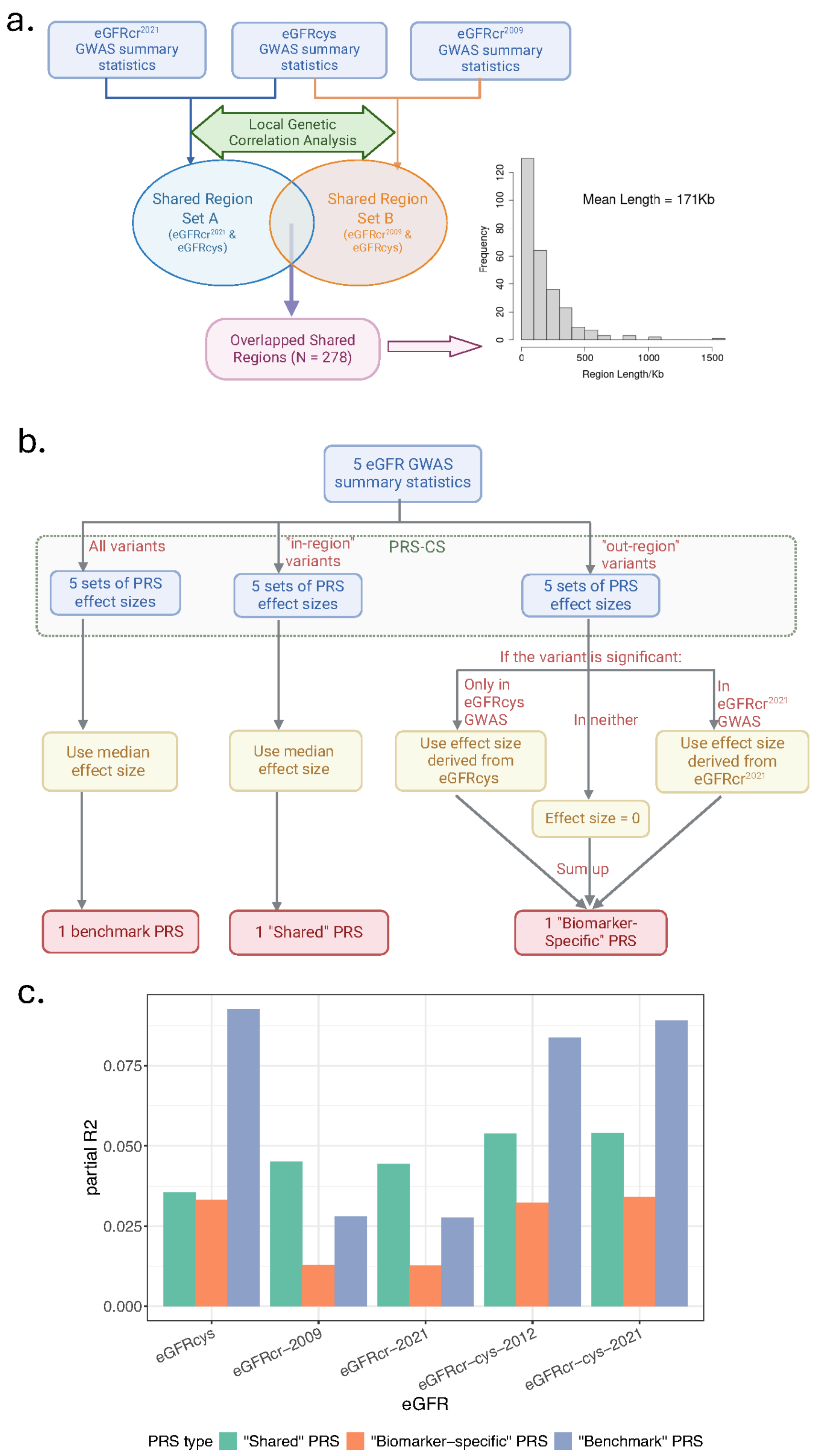

### Figure 2

**Flowchart of genetic correlation analysis and PRS construction. a.**Local genetic correlation analysis was performed on two pairs of eGFR(eGFRcr$^{2021}$ vs eGFRcys, and eGFRcr$^{2009}$ vs eGFRcys), each generated a set of overlapping regions(Set A and B), the intersection was the shared-region. **b.**Three PRSs were constructed by PRS-CS. 1)Benchmark(left branch): five sets of posterior PRS effect sizes were obtained by taking the GWAS summary statistics of all GWAS variants as input of PRS-CS and a single PRS was constructed taking the median effect size for each variant. 2)"Shared"(middle branch): Constructed as described before, however, the input variants were only those identified in the shared regions. 3)"Biomarker-specific"(right branch): after removing ~81k variants outside the shared regions but with high LD ($R^2 > 0.8$) with any "shared" variants, the remaining variants were used to construct a PRS. Variant effect sizes are based on its significance in either eGFRcys or eGFRcr2021. **c.**The partial $R^2$ of developed PRSs in explaining eGFR values.

## Supplementary Files

This is a list of supplementary files associated with this preprint. Click to download.

- ExtendedDataTables.xlsx
- ExtendedDataFigures.pdf
